# Multi-Objective Optimization of Integrated Civilian-Military Scheduling of Medical Supplies for Epidemic Prevention and Control

**DOI:** 10.3390/healthcare9020126

**Published:** 2021-01-28

**Authors:** Hai-Feng Ling, Zheng-Lian Su, Xun-Lin Jiang, Yu-Jun Zheng

**Affiliations:** 1 College of Field Engineering, Army Engineering University, Nanjing 210007, China; hfling@compintell.cn (H.-F.L.); suzhenglian@compintell.cn (Z.-L.S.); 2 Department of Engineering Technology and Application, Army Infantry College, Nanchang 330100, China; xunlinjiang@163.com; 3 School of Information Science and Engineering, Hangzhou Normal University, Hangzhou 311121, China

**Keywords:** medical supplies scheduling, epidemic prevention and control, multi-objective optimization, water wave optimization, civilian-military integration

## Abstract

In a large-scale epidemic, such as the novel coronavirus pneumonia (COVID-19), there is huge demand for a variety of medical supplies, such as medical masks, ventilators, and sickbeds. Resources from civilian medical services are often not sufficient for fully satisfying all of these demands. Resources from military medical services, which are normally reserved for military use, can be an effective supplement to these demands. In this paper, we formulate a problem of integrated civilian-military scheduling of medical supplies for epidemic prevention and control, the aim of which is to simultaneously maximize the overall satisfaction rate of the medical supplies and minimize the total scheduling cost, while keeping a minimum ratio of medical supplies reservation for military use. We propose a multi-objective water wave optimization (WWO) algorithm in order to efficiently solve this problem. Computational results on a set of problem instances constructed based on real COVID-19 data demonstrate the effectiveness of the proposed method.

## 1. Introduction

**Figure 1:**
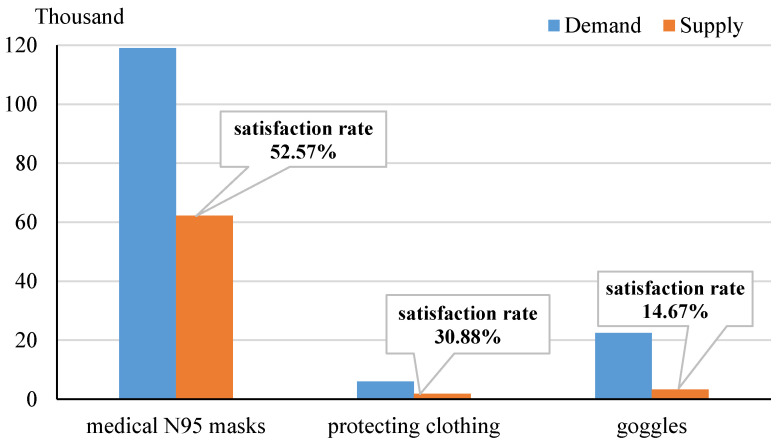
Satisfaction rates to the demands of some medical supplies during the peak period of COVID-19 in Wuhan, China.

A large-scale epidemic outbreak often causes a huge shortage of medical supplies, such as medical masks, protecting clothing, ventilators, sickbeds, and computed tomography (CT), to name just a few [1,2]. For example, during the peak period of the novel coronavirus pneumonia (COVID-19) in Wuhan, China, the satisfaction rates of medical N95 masks, protecting clothing, and goggles are 52.57%, 30.88%, and 14.67%, respectively, as shown in Figure 1. Such a shortage indicates that local civilian medical services are unable to provide sufficient medical supplies to meet the demands that explosively increase during the epidemic. Therefore, scheduling medical supplies from other sources is necessary and critical in the prevention and control the spread of the epidemic. Although logistics in humanitarian disasters, including epidemics, have been extensively studied in the literature [3,4], there exists a research gap in understanding of the impacts of epidemics on supply chains and vice versa [5].

Military medical services are a special class of medical services that are reserved for military use, including servicemen healthcare, field hospitals, international peacekeeping, etc. Under emergency situations, they can provide an effective supplement to the shortage of civilian medical services [6]. However, scheduling medical supplies from military medical services to civilian medical services in a large-scale epidemics has the following difficulties:

- There us often a large number of civilian medical services that are distributed in wide areas, and the shortage of medical supplies is often acute [7,8]; however, the number of military medical services and amount of supplies that they can provide are often limited.
- Using military medical services to support civilian medical services involes not only scheduling medical supplies from the former to the latter, but also scheduling patients from the latter to the former.
- Not all of the supplies of the military medical services can be utilized to support civilian medical services; typically, they must reserve a certain proportion of capabilities for potential military use. Particularly, some military medical services cannot admit outside patients due to military confidentiality requirements.
- The aim is not only to maximize the overall satisfaction rate of medical supplies for epidemic prevention and control, but also to minimize the scheduling cost (a high cost not only indicates a large investment, but is also related to great scheduling efforts and long scheduling time that will significantly decrease the effectiveness of epidemic prevention and control).

Therefore, the problem of integrated civilian-military scheduling of medical supplies for epidemic prevention and control is significantly more complex than those common scheduling problems in commercial supply chains and medical logistics [9,10]. However, in the literature, there are few studies concerning the integration of civilian and military medical supplies in epidemic situations.

Based on the experiences from COVID-19 prevention and control, in this paper we propose an integrated civilian-military medical supply scheduling problem, which is formulated as a complex constrained integer programming problem that is known to be NP-hard [11]. The aim of this problem is to simultaneously maximize the overall satisfaction rate of the medical supplies and minimize the total scheduling cost, while satisfying constraints, including the maximum amount of supplies that can be provided by military medical services, the maximum number of patients that can be received by open military medical services, lower limit of supply satisfaction rate, upper limit of scheduling cost, etc. For large instances of this problem, exact algorithms (such as branch-and-bound) are often impractical. We propose a multi-objective optimization evolutionary algorithm that is based on the water wave optimization (WWO) metaheuristic in order to efficiently solve this difficult problem [12]. We demonstrate the performance advantages of the proposed method as compared to some popular multi-objective optimization algorithms on a set of problem instances that are constructed based on real COVID-19 data in China. The main contributions of this paper can be summarized, as follows:

- We present a problem of scheduling integrated civilian-military medical supplies for the prevention and control of large-scale epidemics, such as COVID-19.
- We propose an efficient multi-objective optimization metaheuristic to solve the problem.
- We demonstrate the performance of the proposed method as compared to state-of-the-arts.

The remainder of this paper is structured, as follows. Section 2 introduces the related work in the literature. Section 3 presents the formulation of the integrated scheduling problem. Section 4 proposes the multi-objective optimization metaheuristic, Section 5 presents the computational results, and Section 6 concludes with a discussion.

## 2. Related Work

In recent years, there are increasing studies on scheduling problems in medical supply chains, which are complex networks that consist of many different parties at various stages [13]. Mete and Zabinsky [14] proposed a stochastic optimization method for the storage and distribution problem of medical supplies for disaster management under a variety of possible disaster types and magnitudes. Their optimization method aims to balance the preparedness and risk under the uncertainties, and its solutions used can suggest the loading and routing of vehicles to transport medical supplies for disaster response. Xu et al. [15] studied an integrated medical supply inventory control system that links demand, service provided at the clinic, health care service provider’s information, inventory storage data, using ABC analysis method, economic order quantity model, two-bin method, and safety stock concept as decision support models. The pilot case study demonstrated that the integrated system holds several advantages for inventory managers Lei et al. [16] studied a problem of personnel scheduling and supplies provisioning in emergency relief operations; they proposed a mathematical programming that is based rolling horizon heuristic that is able to find near-optimal solutions to the problem. Wang et al. [17] modeled an integrated post-disaster medical assistance team scheduling and relief supply distribution problem as a mixed integer-programming problem; they proposed a two-stage hybrid metaheuristic method to solve the problem. Zhang et al. [18] studied a two-stage medical supply chain scheduling problem, and they found that a pseudo-polynomial-time algorithm can solve the problem.

Studies on medical supply chain problems in response to large-scale epidemic situations are relatively few. Queiroz et al. [5] conducted a systematic literature review on supply chains under epidemic outbreaks; their findings suggested that influenza was the most visible epidemic outbreak reported, and that optimization of resource allocation and distribution emerged as the most popular topic. Liu and Zhang [19] presented a dynamic medical logistics model coupling a medical demand forecasting mechanism and a logistics planning system for satisfying the forecasted demand and minimizing the total cost; the problem was formulated as a mixed 0-1 integer programming problem characterizing the decision making at various levels of hospitals, distribution centers, pharmaceutical plants, and the transportation in between them. Büyüktahtakın et al. [20] presented an epidemics-logistics model based on mixed-integer programming that determines the optimal amount, timing, and location of resources to minimize the total number of infections and fatalities under a limited budget over a multi-period planning horizon. They validated the performance of the model using the case of the 2014–2015 Ebola outbreak in Guinea, Liberia, and Sierra Leone. When considering that different diseases have dissimilar diffusion dynamics and can cause different public health emergencies, Liu et al. [21] modified that model by changing capacity constraint, and then applied it to control the 2009 H1N1 outbreak in China. Syahrir et al. [22] used the SEIR model to predict the amount of drug supplies in hospitals during the outbreak of dengue fever, in order to manage and determine the satisfactory amount of drug supplies in the hospital to handle patients who are indicated and infected with dengue quickly and precisely. However, none of the above studies concern the integrated scheduling of civilian and military medical supplies.

## 3. Problem Formulation

Based on the requirements of utilizing both civilian and military medical resources for combating COVID-19 as well as to fill the gap of current researches that are related to this topic, we present a problem of scheduling integrated civilian-military medical supplies for epidemic prevention and control. Formally, we consider that there are *m* civilian medical services that undertake epidemic prevention and control tasks. In the planning horizon (e.g., the next week), the numbers (or expected numbers) of normal residents, suspected cases, mild cases, and severe cases to be served by the *i*-th service are $n_{i}^{o}$, $n_{i}^{s}$, $n_{i}^{m}$, and $n_{i}^{v}$, respectively. The tasks involve *K* types of medical supplies, including $K_{1}$ types of non-fixed supplies (e.g., medical masks, protective clothing, and movable CT) and $K_{2}$ types of fixed supplies (e.g., sickbeds and non-movable CT). We set the non-fixed supplies as the first $K_{1}$ in all *K* supplies. Each type of supply is assigned with a weight $w_{k}$ (1≤k≤K), which is determined according to the importance of the supply in epidemic prevention and control, subject to $\left(\sum_{k=1}^{K}w_{k}\right)=1$. The amount of the *k*-th type of medical supply available at the *i*-th civilian medical service is $a_{ik}$, and the amount the *k*-th type of medical supply required per normal resident, suspected case, mild case, and severe case is $r_{k}^{o}$, $r_{k}^{s}$, $r_{k}^{m}$, and $r_{k}^{v}$, respectively (1≤i≤m;1≤k≤K). Note that the amount can be fractional, e.g., if every 500 suspected cases require one CT per day, then the amount of CT that is required per suspected case is 0.002.

In the large-scale epidemic, most of the supplies that can be provided by the civilian medical services are insufficient. Therefore, we want to utilize the supplies from $m^{\prime }$ military medical services. The amount of the *k*-th type of medical supply available at the *j*-th military medical service is $a_{jk}^{\prime }$ ($1\;\leq \;j\;\leq \;m^{\prime };1\;\leq \;k\;\leq \;K$). Non-fixed supplies can be delivered from military medical services to civilian medical services; however, for fixed supplies, we have to send residents or patients to the locations of military medical services. However, some military medical services cannot admit outside patientsdue to confidentiality requirements. We call such services closed military medical services, and then call the others that can admit outside patients open military medical services Let $m^{\prime \prime }$ be the number of open military medical services, we set them as the first $m^{\prime \prime }$ in all $m^{\prime }$ military medical services ($m^{\prime \prime }\;\leq \;m^{\prime }$).

The problem needs to make decision in three aspects:

(1) Determining the amount of each *k*-th type of non-fixed supply that will be dispatched from each *j*-th military medical service to each *i*-th civilian medical service, as denoted by $x_{ijk}$ ($1\;\leq \;i\;\leq \;m;1\;\leq \;j\;\leq \;m^{\prime };1\;\leq \;k\;\leq \;K_{1}$).
(2) Determining the amount of each *k*-th type of non-fixed supply that will be dispatched from each $j^{\prime }$-th closed military medical service to each *j*-th open military medical service, as denoted by $x_{jj^{\prime }k}^{\prime }$ ($\;1\;\leq \;j\;\leq \;m^{\prime };m^{\prime \prime }\;+1\;\leq \;j^{\prime }\;\leq \;m^{\prime };1\;\leq \;k\;\leq \;K_{1}$).
(3) Determining the numbers of normal residents, suspected cases, mild cases, and severe cases that will be reallocated from each *i*-th civilian medical service to each *j*-th open military medical service, as denoted by $y_{ij}^{o}$, $y_{ij}^{s}$, $y_{ij}^{m}$, and $y_{ij}^{v}$, respectively ($1\;\leq \;i\;\leq \;m;1\;\leq \;j\;\leq \;m^{\prime \prime }$).

The above decision variables are all positive integers. Table 1 lists the input and decision variables of the problem.

### 3.1. Supplies Satisfaction Rates

We can calculate the satisfaction rate of each type of supply at each service, according to the above input and decision variables. For the *i*-th civilian medical service (1≤i≤m), the final numbers of normal residents, suspected cases, mild cases, and severe cases to be served are as follows:

(1) $$\begin{array}{ccc} {\hat{n}}_{i}^{o} & = & n_{i}^{o}-\sum_{j=1}^{m^{\prime \prime }}y_{ij}^{o} \end{array}$$

(2) $$\begin{array}{ccc} {\hat{n}}_{i}^{s} & = & n_{i}^{s}-\sum_{j=1}^{m^{\prime \prime }}y_{ij}^{s} \end{array}$$

(3) $$\begin{array}{ccc} {\hat{n}}_{i}^{m} & = & n_{i}^{m}-\sum_{j=1}^{m^{\prime \prime }}y_{ij}^{m} \end{array}$$

(4) $$\begin{array}{ccc} {\hat{n}}_{i}^{v} & = & n_{i}^{v}-\sum_{j=1}^{m^{\prime \prime }}y_{ij}^{v} \end{array}$$

Subsequeently, the final amount of each *k*-th type of supply that is required by the *i*-th civilian medical service is:

(5) $${\hat{r}}_{ik}={\hat{n}}_{i}^{o}r_{k}^{o}+{\hat{n}}_{i}^{s}r_{k}^{s}+{\hat{n}}_{i}^{m}r_{k}^{m}+{\hat{n}}_{i}^{v}r_{k}^{v}$$

The amount of each type of fixed supply that is available at the civilian medical service does not change, while that of each non-fixed supply is increased by the assistance from military medical services. The final amount of each *k*-th type of supply available at the *i*-th civilian medical service is:

(6) $${\hat{a}}_{ik}=\left\{\begin{array}{cc} a_{ik}+\sum_{j=1}^{m^{\prime }}x_{ijk}, & \;1\;\leq \;k\;\leq \;K_{1}\\ a_{ik}, & \;K_{1}\;+\;1\;\leq \;k\;\leq \;K \end{array}\right.$$

Therefore, the satisfaction rate of the *k*-th type of supply at the *i*-th civilian medical service is:

(7) $$\theta \left(i,k\right)=\min\left(\frac{{\hat{a}}_{ik}}{{\hat{r}}_{ik}},1\right)$$

Next, for the *j*-th open military medical service ($1\;\leq \;j\;\leq \;m^{\prime \prime }$), the numbers of normal residents, suspected cases, mild cases, and severe cases to be served are as follows:

(8) $$\begin{array}{ccc} {\hat{n}}_{j}^{{}^{\prime }o} & = & \sum_{i=1}^{m}y_{ij}^{o} \end{array}$$

(9) $$\begin{array}{ccc} {\hat{n}}_{j}^{{}^{\prime }s} & = & \sum_{i=1}^{m}y_{ij}^{s} \end{array}$$

(10) $$\begin{array}{ccc} {\hat{n}}_{j}^{{}^{\prime }m} & = & \sum_{i=1}^{m}y_{ij}^{m} \end{array}$$

(11) $$\begin{array}{ccc} {\hat{n}}_{j}^{{}^{\prime }v} & = & \sum_{i=1}^{m}y_{ij}^{v} \end{array}$$

Subsequently, the amount of each *k*-th type of supply that is required by the *j*-th open military medical service is:

(12) $${\hat{r}}_{jk}^{\prime }={\hat{n}}_{i}^{{}^{\prime }o}r_{k}^{o}+{\hat{n}}_{i}^{{}^{\prime }s}r_{k}^{s}+{\hat{n}}_{i}^{{}^{\prime }m}r_{k}^{m}+{\hat{n}}_{i}^{{}^{\prime }v}r_{k}^{v}$$

The final amount of each *k*-th type of supply available at the *j*-th open military medical service is:

(13) $${\hat{a}}_{jk}^{\prime }=\left\{\begin{array}{cc} a_{jk}^{\prime }-\sum_{i=1}^{m}x_{ijk}+\sum_{j^{\prime }=m^{\prime \prime }\;+1}^{m^{\prime }}x_{jj^{\prime }k}^{\prime }, & \;1\;\leq \;k\;\leq \;K_{1}\\ a_{jk}^{\prime }, & \;K_{1}\;+\;1\;\leq \;k\;\leq \;K \end{array}\right.$$

Therefore, the satisfaction rate of the *k*-th type of supply at the *j*-th open military medical service is:

(14) $$\theta^{\prime }\left(j,k\right)=\min\left(\frac{{\hat{a}}_{jk}^{\prime }}{{\hat{r}}_{jk}^{\prime }},1\right)$$

The first objective of the problem is to maximize the overall weighted satisfaction rate of all medical supplies, as follows:

(15) $$\max S\left(x,y\right)=\frac{1}{m\;+\;m^{\prime \prime }}\left(\sum_{i=1}^{m}\sum_{k=1}^{K}w_{k}\theta \left(i,k\right)+\sum_{j=1}^{m^{\prime \prime }}\sum_{k=1}^{K}w_{k}\theta^{\prime }\left(i,k\right)\right)$$

### 3.2. Scheduling Costs

Delivering supplies and residents/patients involves costs. We use $c_{ijk}$ to denote the cost of delivering one unit of the *k*-th type of non-fixed supply from the *j*-th military medical service to the *i*-th civilian medical service ($1\;\leq \;i\;\leq \;m;1\;\leq \;j\;\leq \;m^{\prime };1\;\leq \;k\;\leq \;K_{1}$), $c_{jj^{\prime }k}^{\prime }$ to denote the cost of delivering one unit of the *k*-th type of non-fixed supply from the $j^{\prime }$-th closed military medical service to the *j*-th open military medical service ($1\;\leq \;j\;\leq \;m^{\prime \prime };m^{\prime \prime }\;+\;1\;\leq \;j^{\prime }\;\leq \;m^{\prime };1\;\leq \;k\;\leq \;K_{1}$), and $c_{ij}^{o}$, $c_{ij}^{s}$, $c_{ij}^{m}$, and $c_{ij}^{v}$ to denote the cost of delivering one normal resident, suspected case, mild case, and severe case from the *i*-th civilian medical service to the *j*-th open military medical service, respectively ($1\;\leq \;i\;\leq \;m;1\;\leq \;j\;\leq \;m^{\prime \prime }$).

*Remark:* in practice, the delivery cost is not linearly proportional to the amount of supplies or number of patients. We make this assumption to simplify the cost computation. For example, suppose that the cost of scheduling a vehicle from the *j*-th military medical service to the *i*-th civilian medical service is 100, a unit of the *k*-th type of supply occupies two percent of the volume of the vehicle, then we set $c_{ijk}\;=\;5$. Although the cost is subject to variation (e.g., the last vehicle is often not fully loaded), we neglect such a variation because of the large amounts of supplies to be delivered.

The total scheduling cost of a solution (x,y) is:

(16) $$C\left(x,y\right)=\sum_{i=1}^{m}\sum_{j=1}^{m^{\prime }}\sum_{k=1}^{K_{1}}c_{ijk}x_{ijk}+\sum_{j=1}^{m^{\prime \prime }}\sum_{j^{\prime }=m^{\prime \prime }\;+\;1}^{m^{\prime }}\sum_{k=1}^{K_{1}}c_{ijk}^{\prime }x_{ijk}^{\prime }+\sum_{i=1}^{m}\sum_{j=1}^{m^{\prime }}\left(c_{ij}^{o}y_{ij}^{o}+c_{ij}^{s}y_{ij}^{s}+c_{ij}^{m}y_{ij}^{m}+c_{ij}^{v}y_{ij}^{v}\right)$$

The second objective of the problem is to minimize the total scheduling cost. In practice, the decision-maker typically sets an upper limit $\bar{C}$ for the total scheduling cost and a lower limit $\underline{S}$ for the overall supply satisfaction rate. We utilize $\bar{C}$ to transform the second objective to scale the second objective to the same order of magnitude as the first objective, as follows:

(17) $$\max C^{\prime }\left(x,y\right)=1-\frac{\min(C\left(x,y\right),\bar{C})}{2\bar{C}}$$

### 3.3. Constraints

A solution (x,y) to the problem must satisfy the following constraints.

- A military medical service must reserve a minimum amount $b_{jk}$ of each type of supply (e.g., for unexpected military use):
  (18) $$\begin{array}{ccc} a_{jk}^{\prime }-\sum_{i=1}^{m}x_{ijk}+\sum_{j^{\prime }=m^{\prime \prime }\;+\;1}^{m^{\prime }}x_{jj^{\prime }k}^{\prime } & \geq & b_{jk},\;1\;\leq \;j\;\leq \;m^{\prime \prime };1\;\leq \;k\;\leq \;K_{1} \end{array}$$
  (19) $$\begin{array}{ccc} a_{jk}^{\prime }-\sum_{i=1}^{m}x_{ijk}-\sum_{j^{\prime }=1}^{m^{\prime \prime }}x_{j^{\prime }jk}^{\prime } & \geq & b_{jk},\;m^{\prime }\;-\;m^{\prime \prime }\;+\;1\;\leq \;j\;\leq \;m^{\prime };1\;\leq \;k\;\leq \;K_{1} \end{array}$$
  (20) $$\begin{array}{ccc} a_{jk}^{\prime }-\sum_{i=1}^{m}y_{ij}^{o}r_{k}^{o}-\sum_{i=1}^{m}y_{ij}^{s}r_{k}^{s}-\sum_{i=1}^{m}y_{ij}^{m}r_{k}^{m}-\sum_{i=1}^{m}y_{ij}^{v}r_{k}^{v} & \geq & b_{jk},\;1\;\leq \;j\;\leq \;m^{\prime \prime };K_{1}\;+\;1\;\leq \;k\;\leq \;K \end{array}$$
- The number of residents/patients received by an open military medical service has an upper limit (denoted by an overline):
  (21) $$\begin{array}{ccc} \sum_{i=1}^{m}y_{ij}^{o} & \leq & {\bar{n}}_{j}^{o},\;1\;\leq \;j\;\leq \;m^{\prime \prime } \end{array}$$
  (22) $$\begin{array}{ccc} \sum_{i=1}^{m}y_{ij}^{s} & \leq & {\bar{n}}_{j}^{s},\;1\;\leq \;j\;\leq \;m^{\prime \prime } \end{array}$$
  (23) $$\begin{array}{ccc} \sum_{i=1}^{m}y_{ij}^{m} & \leq & {\bar{n}}_{j}^{m},\;1\;\leq \;j\;\leq \;m^{\prime \prime } \end{array}$$
  (24) $$\begin{array}{ccc} \sum_{i=1}^{m}y_{ij}^{v} & \leq & {\bar{n}}_{j}^{v},\;1\;\leq \;j\;\leq \;m^{\prime \prime } \end{array}$$
- The number of residents/patients sent from a civilian medical service cannot be larger than the current number:
  (25) $$\begin{array}{ccc} \sum_{j=1}^{m^{\prime }}y_{ij}^{o} & \leq & n_{i}^{o},\;1\;\leq \;i\;\leq \;m \end{array}$$
  (26) $$\begin{array}{ccc} \sum_{j=1}^{m^{\prime }}y_{ij}^{s} & \leq & n_{i}^{s},\;1\;\leq \;i\;\leq \;m \end{array}$$
  (27) $$\begin{array}{ccc} \sum_{j=1}^{m^{\prime }}y_{ij}^{m} & \leq & n_{i}^{m},\;1\;\leq \;i\;\leq \;m \end{array}$$
  (28) $$\begin{array}{ccc} \sum_{j=1}^{m^{\prime }}y_{ij}^{v} & \leq & n_{i}^{v},\;1\;\leq \;i\;\leq \;m \end{array}$$
- The overall supply satisfaction rate cannot be below the low limit:
  (29) $$S\left(x,y\right)\geq \underline{S}$$
- The total scheduling cost cannot exceed the upper limit:
  (30) $$C\left(x,y\right)\leq \bar{C}$$

## 4. Method

The above problem is a bi-objective constrained integer programming problem. It is known to be NP-hard, even if only one objective is retained [11]. In a large-scale epidemic, the number of services, number of types of supplies, and number of residents/patients are all often very large and, thus, it is impractical to use traditional exact algorithms, such as branch-and-bound [23], to solve such instances within a reasonable computational time.

We propose a multi-objective WWO (MOWWO) algorithm, which is capable of obtaining a near Pareto-optimal front within a short response time, in order to efficiently solve the problem. WWO is metaheuristic borrowing principles from the shallow water wave theory to solve optimization problems [12]. WWO evolves a population of solutions, each having a wavelength λ that is inversely proportional to its fitness. At each generation, each solution produces a child solution in the hyper-sphere with a radius of λ, such that high-fitness solutions exploit small areas around them, while low-fitness solutions explore large areas in the solution space, so as to balance global and local search. WWO also performs an intensive local search around a newly found best solution, and it violates a solution if it fails to generate a better child after a specified number of generations to avoid search stagnation.

### 4.1. Solution Initialization

For the considered medical supplies scheduling problem, we create a population of solutions by initializing each solution while using the following steps:

(1) For each *i*-th civilian medical service, determine a percent $p_{i}$, such that its current available supplies (including human resources and other hardware and software resources) are just sufficient to treat $p_{i}n_{i}^{o}$ normal residents, $p_{i}n_{i}^{s}$ suspected cases, $p_{i}n_{i}^{m}$ mild cases, and $p_{i}n_{i}^{v}$ severe cases (1≤i≤m);
(2) If $p_{i}$ is less than 100% (which indicates that the civilian medical service cannot serve all of the residents/patients allocated to it), set a random ratio $\gamma_{i}^{o}\in \left[0,1\right]$ of normal residents that will be sent to open military medical services, and randomly divide $\left(1\;-\;p_{i}\right)\gamma_{i}^{o}n_{i}^{o}$ into $m^{\prime \prime }$ parts to obtain decision variables $y_{ij}^{o}$ ($1\;\leq \;i\;\leq \;m;1\;\leq \;j\;\leq \;m^{\prime \prime }$);
(3) Similarly, set random ratios $\gamma_{i}^{s}$, $\gamma_{i}^{m}$, $\gamma_{i}^{v}$ of suspected cases, mild cases, and severe cases that will be sent to open military services, and randomly divide each of $\left(1\;-\;p_{i}\right)\gamma_{i}^{s}n_{i}^{s}$, $\left(1\;-\;p_{i}\right)\gamma_{i}^{m}n_{i}^{m}$, and $\left(1\;-\;p_{i}\right)\gamma_{i}^{v}n_{i}^{v}$ into $m^{\prime \prime }$ parts to obtain decision variables $y_{ij}^{s}$, $y_{ij}^{m}$, and $y_{ij}^{v}$, respectively ($1\;\leq \;i\;\leq \;m;1\;\leq \;j\;\leq \;m^{\prime \prime }$);
(4) For each *k*-th type of non-fixed supply, calculate the set $C_{k}$ of civilian medical services, where the supply is not sufficient for treating the residents/patients remaining at the services ($1\;\leq \;k\;\leq \;K_{1}$);
(5) For each *j*-th open military medical service and each *k*-th type of non-fixed supply, if the supply is sufficient for the residents/patients that are received by the service, divide the remaining amount of this supply into $|C_{k}|$ parts for the civilian medical services to obtain $|C_{k}|$ decision variables $x_{ijk}$, and set $x_{ijk}\;=\;0$ for other $i\notin C_{k}$ ($1\;\leq \;i\;\leq \;m;1\;\leq \;j\;\leq \;m^{\prime \prime };1\;\leq \;k\;\leq \;K_{1}$);
(6) For each *k*, update $C_{k}$ by removing those civilian medical services that receive sufficient *k*-th type of supply ($1\;\leq \;k\;\leq \;K_{1}$);
(7) For each *k*, calculate the set $O_{k}$ of open military medical services, where the *k*-th type of supply is not sufficient to treat the residents/patients received at the services ($1\;\leq \;k\;\leq \;K_{1}$);
(8) For each $j^{\prime }$-th closed military medical service and each *k*-th type of non-fixed supply, divide the amount $\left(a_{jk}^{\prime }-b_{jk}\right)$ of this supply into $|C_{k}\cup D_{k}|$ parts for the civilian and open military medical services to obtain $|C_{k}\cup D_{k}|$ decision variables $x_{jj^{\prime }k}^{\prime }$, and set $x_{jj^{\prime }k}^{\prime }\;=\;0$ for other $i\notin C_{k}$ and $j\notin D_{k}$ ($1\;\leq \;j\;\leq \;m^{\prime \prime };m^{\prime \prime }\;+1\;\leq \;j^{\prime }\;\leq \;m^{\prime };1\;\leq \;k\;\leq \;K_{1}$).

### 4.2. Solution Evolution

The original WWO is for single-objective optimization. Here, we extend it for multi-objective optimization. First, we employ the fast non-dominated sorting procedure of NSGA-II [24] to compute a rank rank(X) for each solution X=(x,y) in the population. That is, the rank of each non-dominated solution in the population is 1; afterwards, these current non-dominated solutions are temporarily excluded from the population and the rank of each new non-dominated solution is 2; this procedure continues until all of the solutions are ranked. Any infeasible solution that violates a constraint is considered to be dominated by all feasible solutions. According to the rank of each solution *X*, we calculate its wavelength as:

(31) $$\lambda \left(X\right)=\alpha \cdot \frac{\mathrm{rank}(X)}{{\mathrm{rank}}_{\max}}$$

where ${\mathrm{rank}}_{\max}$ is the maximum rank number among the population and α is a control parameter.

At each generation, each solution *X* produces a child solution while using the following procedure:

(1) For each *i*-th civilian medical service (1≤i≤m), try $m^{\prime \prime }/2$ times, each of which performs one of the following three operations with a probability of λ(X):
  (1.1) randomly select a *j*-th open military medical service with $y_{ij}^{o}\;>\;0$ ($1\;\leq \;j\;\leq \;m^{\prime }$), let $\Delta =\mathrm{rand}(0,y_{ij}^{o})$, and set $y_{ij}^{o}=y_{ij}^{o}\;-\;\Delta$;
  (1.2) randomly select two different *j*-th and $j^{\prime }$-th open military medical services ($1\;\leq \;j,j^{\prime }\;\leq \;m^{\prime }$), let $\Delta =\mathrm{rand}(0,\min\left(y_{ij}^{o},y_{ij^{\prime }}^{o}\right))$, set $y_{ij}^{o}=y_{ij}^{o}\;+\;\Delta$ and $y_{ij^{\prime }}^{o}=y_{ij^{\prime }}^{o}\;-\;\Delta$; and,
  (1.3) if $\gamma_{i}^{o}>0$, let $\Delta =\mathrm{rand}(0,\gamma_{i}^{o}n_{ij}^{o})$, randomly select a *j*-th open military medical service ($1\;\leq \;j\;\leq \;m^{\prime }$), and set $y_{ij}^{o}=y_{ij}^{o}\;+\;\Delta$.
(2) For each *i*-th civilian medical service (1≤i≤m), try $m^{\prime \prime }/2$ times, each of which performs operations similar to the above to change $y_{ij}^{s}$.
(3) For each *i*-th civilian medical service (1≤i≤m), try $m^{\prime \prime }/2$ times, each of which performs operations that are similar to the above to change $y_{ij}^{m}$.
(4) For each *i*-th civilian medical service (1≤i≤m), try $m^{\prime \prime }/2$ times, each of which performs operations similar to the above to change $y_{ij}^{v}$.
(5) If the fitness of *X* is in the second half of the population, use solution initialization steps 4)–8) to reset the other components of the solution, and then stop the procedure.
(6) Otherwise, for each *j*-th open military medical service and each *k*-th non-fixed supply ($1\;\leq \;j\;\leq \;m^{\prime \prime };1\;\leq \;k\;\leq \;K_{1}$), try m/2 times, each of which, with a probability of λ(X), randomly select two different *i*-th and $i^{\prime }$-th civilian medical services ($1\;\leq \;i,i^{\prime }\;\leq \;m$), let $\Delta =\mathrm{rand}(0,\min\left(x_{ijk},x_{i^{\prime }jk}\right))$, set $x_{ijk}=x_{ijk}\;+\;\Delta$ and $x_{i^{\prime }jk}=x_{i^{\prime }jk}\;-\;\Delta$.
(7) For each $j^{\prime }$-th closed military medical service and each *k*-th non-fixed supply ($m^{\prime }\;-\;m^{\prime \prime }\;+\;1\;\leq \;j^{\prime }\;\leq \;m^{\prime \prime };1\;\leq \;k\;\leq \;K_{1}$), try $(m\;+\;m^{\prime \prime })/2$ times, each of which, with a probability of λ(X), randomly select an *i*-th civilian medical service and an $i^{\prime }$-th civilian or a *j*-th open military medical services ($1\;\leq \;i,i^{\prime }\;\leq \;m;1\;\leq \;j\;\leq \;m^{\prime \prime }$), let $\Delta =\mathrm{rand}(0,\min\left(x_{ij^{\prime }k},x_{i^{\prime }j^{\prime }k}\right))$ or $\Delta =\mathrm{rand}(0,\min\left(x_{ij^{\prime }k},x_{jj^{\prime }k}^{\prime }\right))$, set $x_{ij^{\prime }k}=x_{ij^{\prime }k}\;\pm \;\Delta$ and $x_{i^{\prime }j^{\prime }k}=x_{i^{\prime }j^{\prime }k}\;\mp \;\Delta$ or $x_{jj^{\prime }k}^{\prime }=x_{jj^{\prime }k}\;\mp \;\Delta$.

The above procedure may produce an infeasible solution that violates Constraints (18)–(24), (29) and (30), but Constraints (25)–(28) are always kept satisfied. We try to repair an infeasible solution, as follows:

- If Constraint (18) is violated, we continually select a random *i* with $x_{ijk}\;>\;0$ and decrease $x_{ijk}$ by one until the remaining amount of the *k*-th type of supply in the *j*-th open military medical service is equal to $b_{jk}$;
- If Constraint (19) is violated, then we continually select a random *i* with $x_{ijk}\;>\;0$ or $j^{\prime }$ with $x_{j^{\prime }jk}^{\prime }\;>\;0$ and decrease $x_{ijk}$ or $x_{j^{\prime }jk}^{\prime }$ by one until the remaining amount of the *k*-th type of supply in the *j*-th closed military medical service is equal to $b_{jk}$;
- If Constraint (21) is violated, we continually select a random *i* with $y_{ij}^{o}\;>\;0$ and decrease $y_{ij}^{o}$ by one until the number of residents/patients that are received by the *j*-th open military medical service is equal to $\bar{n}o_{j}$;
- Similarly, if Constraint (22) or (23) or (24) is violated, then we continually select a random *i* and decrease the number of patients sent from the *i*-th civilian medical service to the *j*-th military medical service by one until the constraint is satisfied.

Therefore, after repairing, only Constraints (19), (29), and (30) may be violated, and, in such a case, the solution has the maximum rank in the population.

We compare two solution, as follows: a solution *X* is considered to be better than another solution $X^{\prime }$, if the rank of *X* is smaller than that of $X^{\prime }$, or *X* and $X^{\prime }$ have the same rank, but the number of solutions in the population that are dominated by *X* is larger than that are dominated by $X^{\prime }$. If a child solution is better than its parent, it will replace its parent in the population.

Whenever finding a new solution $X^{*}$ that dominates all of the current non-dominated solutions, the algorithm performs extensive local search around $X^{*}$ to produce $K_{N}$ neighboring solutions (where $K_{N}$ is a control parameter), each of which are obtained by randomly selecting a dimension and set the corresponding component $y_{ij}^{o}{=}_{ij}^{o}\pm 1$, $y_{ij}^{s}{=}_{ij}^{s}\pm 1$, $y_{ij}^{m}{=}_{ij}^{m}\pm 1$, $y_{ij}^{v}{=}_{ij}^{v}\pm 1$, $x_{ijk}=x_{ijk}\pm 1$, or $x_{jj^{\prime }k}^{\prime }=x_{jj^{\prime }k}\pm 1$ (the minus operation can only be performed when minuend is positive). If a neighbor is infeasible, we also repair it using the above steps. Among $X^{*}$ and its $K_{N}$ neighbors, the best one is retained in the population.

### 4.3. Algorithm Framework

Algorithm 1 presents the framework of the MOWWO algorithm for the integrated civilian-military medical supply scheduling problem. The current non-dominated solution set is returned when the algorithm stops. We present the set to the decision-maker with an illustration of the objective function value distribution, and the decision-maker selects a final solution for implementation according to his/her preference on supply satisfaction rate and scheduling cost. 
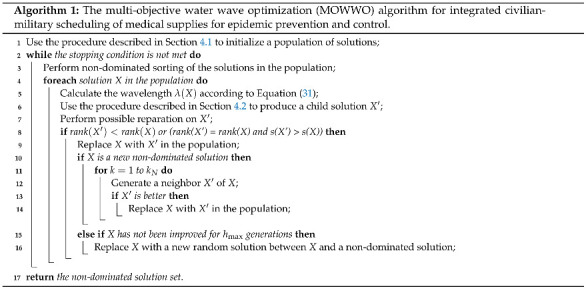


## 5. Results and Discussion

We test the proposed method on four problem instances that are constructed based on data from three cities during January and February, 2020, the peak period of COVID-19 epidemic in China. Table 2 summarizes the main characteristics of the instances. In order to validate the performance of the MOWWO algorithm, we compare it with the following popular multi-objective optimization algorithms:

- The improved fast non-dominated genetic algorithm (NSGA-II) [24].
- The strength Pareto evolutionary algorithm (SPEA2) [25].
- The multi-objective biogeography-based optimization (MOBBO) algorithm [26].
- The multi-objective particle swam optimization (MOPSO) algorithm [27].
- The multi-objective artificial bee colony (MOABC) algorithm [28].

We tune the parameters of all the algorithms on the four instances. Due to the emergency of medical supplies scheduling, we set the maximum CPU time to 600 s as the stop condition for each algorithm. The computational environment is a workstation with an i7-6500 2.5GH CPU, 8GB DDR4 RAM, and an NVIDIA Quadro M500M card. On each instance, we perform 50 Monte Carlo simulation runs of each algorithm. Among the 50 runs, we calculate the best aggregated objective function value that was obtained by each algorithm, as follows:

(32) $$\max f\left(X\right)=wS\left(X\right)+\left(1\;-\;w\right)C^{\prime }\left(X\right)$$

where the aggregation weight *w* is respectively set to 0, 0.1, 0.2, ⋯, 1.0.

Based on non-dominated solution set that was obtained by the algorithms, we also calculate the following metrics according to the hyper-volume (where the reference point is set to $S\left(X\right)=\underline{S}$ and $C^{\prime }\left(X\right)=0.5$) and coverage indicators [29,30] for each comparative algorithm over the 50 runs:

- $R_{H}\left(S_{N},S_{N}^{\prime }\right)$, the ratio of the hyper-volume $H_{V}$ of the non-dominated solution set $S_{N}$ of MOWWO to that of the resulting solution set $S_{N}^{\prime }$ of the comparative algorithm:
  (33) $$R_{H}\left(S_{N},S_{N}^{\prime }\right)=\frac{H_{V}\left(S_{N}\right)}{H_{V}\left(S_{N}^{\prime }\right)}$$
- $C_{V}\left(S_{N},S_{N}^{\prime }\right)$, the fraction of $S_{N}^{\prime }$ that are strictly dominated by at least one non-dominated solution in $S_{N}$ (where ≻ denotes the strict dominance relation):
  (34) $$C_{V}\left(S_{N},S_{N}^{\prime }\right)=\frac{\left|\{X^{\prime }\in S_{N}^{\prime }|\exists X\in S_{N}:X≻X^{\prime }\}\right|}{|S_{N}^{\prime }|}\times 100\%$$
- $C_{V}^{\prime }\left(S_{N}^{\prime },S_{N}\right)$, the fraction of $S_{N}$ that are strictly dominated by at least one non-dominated solution in $S_{N}^{\prime }$:
  (35) $$C_{V}^{\prime }\left(S_{N}^{\prime },S_{N}\right)=\frac{\left|\{X\in S_{N}|\exists X^{\prime }\in S_{N}^{\prime }:X^{\prime }≻X\}\right|}{|S_{N}|}\times 100\%$$

**Figure 2:**
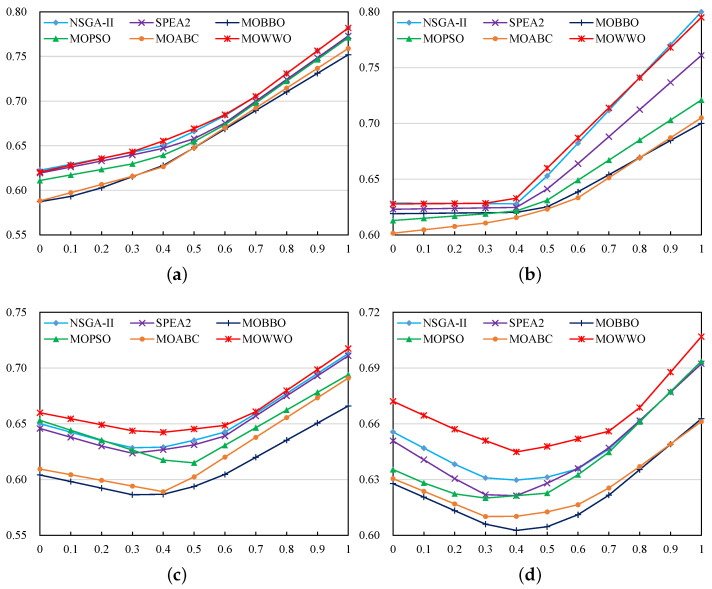
Comparison of the aggregated objective function values obtained by the six algorithms on the four problem instances. The horizontal axis denotes the weight of supply satisfaction rate, and the vertical axis denotes the aggregated objective function value. (**a**) Instance 1; (**b**) Instance 2; (**c**) Instance 3; (**d**) Instance 4.

Table 3 presents the above metric values of the comparative algorithm on the four problem instances, and Figure 2a–d compare the best aggregated objective function values that are obtained by the different algorithms on the four instances, respectively. The proposed MOWWO algorithm has significant performance advantages over the comparative algorithms on all four instances, as it can be observed from the results. On the smallest-size instance 1, the solution set of MOWWO covers the whole solution sets of MOBBO and MOABC, covers a majority of solutions of SPEA-2 and MOPSO, and covers a small fraction of solutions of NSGA-II; on the contrary, none of the solutions of MOWWO are strictly dominated by those of the other algorithms. On instance 2, the solution set of MOWWO covers the whole solution sets of MOBBO, MOPSO, and MOABC, and it covers 22.2% and 62.5% of those of NSGA-II and SPEA2, respectively. On instance 3, the solution set of MOWWO covers the whole solution sets of MOBBO, MOPSO, and MOABC, and covers a majority of those of NSGA-II and SPEA2. On the largest-size instance 4, the solution set of MOWWO covers the whole solution sets of all other algorithms. Additionally, on these instances, none of the solutions of MOWWO are strictly dominated by those of the other algorithms.

Moreover, on each of the instances, the hyper-volume of the solution set of MOWWO is always larger than those of the other four algorithms. In almost all cases, the aggregated objective function value of MOWWO is the best among all of the algorithms. This indicates that MOWWO can provide the most prominent solutions to the decision-maker no matter which preference he/she has (e.g., has more preference to high satisfaction rate or low scheduling cost or a balance between them). The results demonstrates that, among the six algorithms, our MOWWO algorithm exhibits the best performance in solving the test instances of the considered integrated civilian-military medical supply scheduling problem for epidemic prevention and control.

**Figure 3:**
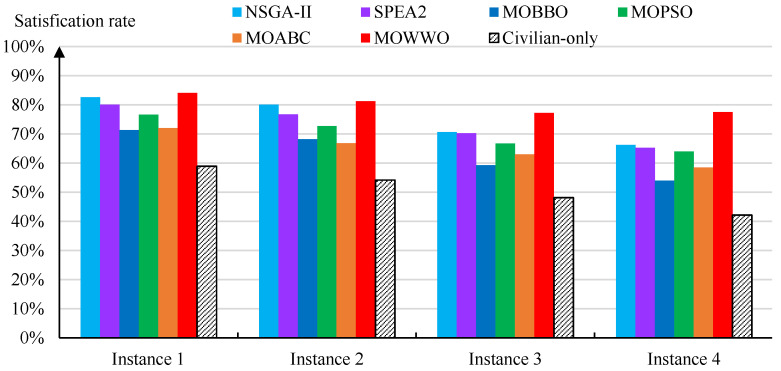
Satisfaction rates that were obtained by the six algorithms as well as a mixed 0–1 integer programming approach [19] (only using civilian medical supplies) on the test instances.

In order to validate the effectiveness of the methods, on each instance, we also evaluate the overall weighted satisfaction rate of all medical supplies that are obtained by the solution of each of the six algorithms as well as the solution of mixed 0–1 integer programming [19] that only uses civilian medical supplies. The weight of each type of supply is determined by public health experts according to its importance in epidemic control. Figure 3 presents the resulting satisfaction rates, which show that the integrated civilian-military medical resource scheduling methods achieve significantly higher satisfaction rates than the pure civilian medical resource scheduling method; among the six algorithms used for integrated civilian-military scheduling, our MOWWO algorithm also always achieves the highest satisfaction rate on each instance. In particular, the overall satisfaction rates that are obtained by MOWWO are 143%, 150%, 160%, and 184% of those of the pure civilian scheduling method on the four instances, respectively. Such high satisfaction rate improvements can significantly contribute to the effectiveness of the prevention and control of the epidemic.

Finally, we summarize a general procedure, as follows, for applying the proposed method in epidemic control:

(1) collect information regarding the demands and available amounts of different medical supplies for all civilian medical services; when estimating the demands, we should consider demand variations in the near future according to the dynamic changes of the epidemic [31,32];
(2) collect information regarding potential military medical services that can provide medical assistances, and then decide which military medical services are selected;
(3) construct an instance of the problem based on the data collected;
(4) apply the proposed algorithm or other algorithms to solve the problem instance, and present the solutions with preference illustration to the decision-maker; and,
(5) monitor the implementation of the final solution; if the implementation deviates from the calculation, or new requirement comes, adjust the model and/or solution to better meet the new situation.

## 6. Conclusions

This paper presents an integrated civilian-military medical supply scheduling problem, the aim of which is to simultaneously maximize the overall satisfaction rate of the medical supplies and minimize the total scheduling cost, while satisfying constraints, including the maximum amount of supplies that can be provided by military medical services, the maximum number of patients that can be received by open military medical services, lower the limit of supply satisfaction rate, upper limit of scheduling cost, etc. In order to efficiently solve this difficult problem, we propose an MOWWO algorithm, which exhibits significant performance advantages when compared to some popular multi-objective optimization algorithms on a set of problem instances constructed based on real COVID-19 data in China.

In the domain of emergency management, the integrated scheduling of civilian and military resources is a common paradigm, and our model can be applied to similar problems in other mass disasters, such as earthquakes, typhoons, and chemical explosions. Although they typically involve more types of supplies, the underlying mathematical model does not differ significantly. Theoretically, our problem can be generalized to an abstract model of integrated scheduling resources from two or more heterogeneous sources to support a common task. It covers a wide range of practical problems, such as integrated scheduling of civilian and military transportation capacities for troop delivery [33], integrated scheduling of in-school and out-school resources for course teaching [34], and integrated scheduling of governmental and nongovernmental rescue teams for disaster rescue [35]. Subsequently, the proposed algorithm can be easily adapted or extended to efficiently solve these problems.

Currently, the problem formulation assumes that the number of residents/patients for each civilian medical service is exact. In practice, such numbers are roughly estimated, while the model and solution quality are sensitive to the accuracies of such numbers. Therefore, our ongoing study is to incorporate mathematical models (e.g., [36,37]) in order to predict the spread of the epidemic and consequent supply demands in a more accurate manner. Our future work will also integrate the scheduling of vehicle for supply delivery [38] and the scheduling of supply production [2] into our approach in order to provide a more comprehensive decision support for epidemic prevention and control.

## Figures and Tables

**Table 1:** Input and decision variables used in the problem formulation.

Symbol	Description
*m*	Number of civilian medical services
$m^{\prime }$	Number of military medical services
$m^{\prime \prime }$	Number of open military medical services
$n_{i}^{o}$	Number of normal residents in the *i*-th civilian medical service (1≤i≤m)
$n_{i}^{s}$	Number of suspected cases in the *i*-th civilian medical service (1≤i≤m)
$n_{i}^{m}$	Number of mild cases in the *i*-th civilian medical service (1≤i≤m)
$n_{i}^{v}$	Number of severe cases in the *i*-th civilian medical service (1≤i≤m)
*K*	Number of medical supplies
$K_{1}$	Number of non-fixed medical supplies
$K_{2}$	Number of fixed medical supplies
$w_{k}$	Importance weight of the *k*-th supply in epidemic prevention and control (1≤k≤K)
$r_{k}^{o}$	Amount the *k*-th supply required per normal resident (1≤k≤K)
$r_{k}^{s}$	Amount the *k*-th supply required per suspected case (1≤k≤K)
$r_{k}^{m}$	Amount the *k*-th supply required per mild case (1≤k≤K)
$r_{k}^{v}$	Amount the *k*-th supply required per severe case (1≤k≤K)
$a_{ik}$	Amount the *k*-th medical supply available at the *i*-th civilian medical service (1≤i≤m;1≤k≤K)
$a_{jk}^{\prime }$	Amount the *k*-th medical supply available at the *j*-th military medical service ($1\;\leq \;j\;\leq \;m^{\prime };1\;\leq \;k\;\leq \;K$)
$c_{ijk}$	Cost of delivering one unit of the *k*-th supply from the *j*-th military medical service to the *i*-th civilian medical service ($1\;\leq \;i\;\leq \;m;1\;\leq \;j\;\leq \;m^{\prime };1\;\leq \;k\;\leq \;K_{1}$)
$c_{jj^{\prime }k}^{\prime }$	Cost of delivering one unit of the *k*-th type supply from the $j^{\prime }$-th closed military medical service to the *j*-th open military medical service ($1\;\leq \;j\;\leq \;m^{\prime \prime };m^{\prime \prime }\;+\;1\;\leq \;j^{\prime }\;\leq \;m^{\prime };1\;\leq \;k\;\leq \;K_{1}$)
$c_{ij}^{o}$	Cost of delivering one normal resident from the *i*-th civilian medical service to the *j*-th military medical service ($1\;\leq \;i\;\leq \;m;1\;\leq \;j\;\leq \;m^{\prime \prime }$)
$c_{ij}^{s}$	Cost of delivering one suspected case from the *i*-th civilian medical service to the *j*-th military medical service ($1\;\leq \;i\;\leq \;m;1\;\leq \;j\;\leq \;m^{\prime \prime }$)
$c_{ij}^{m}$	Cost of delivering one mild case from the *i*-th civilian medical service to the *j*-th military medical service ($1\;\leq \;i\;\leq \;m;1\;\leq \;j\;\leq \;m^{\prime \prime }$)
$c_{ij}^{v}$	Cost of delivering one severe case from the *i*-th civilian medical service to the *j*-th military medical service ($1\;\leq \;i\;\leq \;m;1\;\leq \;j\;\leq \;m^{\prime \prime }$)
$b_{jk}$	Minimum amount of *k*-th supply that must be reserved at the *j*-th military medical service ($1\;\leq \;j\;\leq \;m^{\prime };1\;\leq \;k\;\leq \;K$)
${\bar{n}}_{j}^{o}$	Maximum number of normal residents that can be received by the *j*-th military medical service ($1\;\leq \;j\;\leq \;m^{\prime \prime }$)
${\bar{n}}_{j}^{s}$	Maximum number of suspected cases that can be received by the *j*-th military medical service ($1\;\leq \;j\;\leq \;m^{\prime \prime }$)
${\bar{n}}_{j}^{m}$	Maximum number of mild cases that can be received by the *j*-th military medical service ($1\;\leq \;j\;\leq \;m^{\prime \prime }$)
${\bar{n}}_{j}^{v}$	Maximum number of severe cases that can be received by the *j*-th military medical service ($1\;\leq \;j\;\leq \;m^{\prime \prime }$)
$\bar{C}$	Upper limit of the total scheduling cost
$\underline{S}$	Lower limit of the overall supply satisfaction rate
$x_{ijk}$	Amount of the *k*-th supply that will be dispatched from the *j*-th military medical service to the *i*-th civilian medical service
$x_{jj^{\prime }k}^{\prime }$	Amount of the *k*-th supply that will be dispatched from the $j^{\prime }$-th closed military medical service to the *j*-th open military medical service
$y_{ij}^{o}$	Number of normal residents that will be reallocated from each *i*-th civilian medical service to each *j*-th military medical service ($1\;\leq \;i\;\leq \;m;1\;\leq \;j\;\leq \;m^{\prime \prime }$)
$y_{ij}^{s}$	Number of suspected cases that will be reallocated from each *i*-th civilian medical service to each *j*-th military medical service ($1\;\leq \;i\;\leq \;m;1\;\leq \;j\;\leq \;m^{\prime \prime }$)
$y_{ij}^{m}$	Number of mild cases that will be reallocated from each *i*-th civilian medical service to each *j*-th military medical service ($1\;\leq \;i\;\leq \;m;1\;\leq \;j\;\leq \;m^{\prime \prime }$)
$y_{ij}^{v}$	Number of severe cases that will be reallocated from each *i*-th civilian medical service to each *j*-th military medical service ($1\;\leq \;i\;\leq \;m;1\;\leq \;j\;\leq \;m^{\prime \prime }$)

**Table 2:** Summary of the main characteristics of the four problem instances, where $\sum_{i}n_{i}^{o}$, $\sum_{i}n_{i}^{s}$, $\sum_{i}n_{i}^{m}$, and $\sum_{i}n_{i}^{v}$ denote the total numbers of normal residents, suspected cases, mild cases, and severe cases, respectively.

ID	*m*	$m^{\prime }$	$m^{\prime \prime }$	$\sum_{i}n_{i}^{o}$	$\sum_{i}n_{i}^{s}$	$\sum_{i}n_{i}^{m}$	$\sum_{i}n_{i}^{v}$	$K_{1}$	$K_{2}$
1	39	5	3	1,602,112	1556	385	132	12	7
2	42	6	4	3,195,823	2094	297	96	12	9
3	58	8	5	6,857,710	6720	2335	416	11	8
4	58	8	6	10,693,117	8164	3566	601	13	10

**Table 3:** Comparison of the results of MOWWO with those of the other five algorithms on the four problem instances.

ID	Metrics	NSGA-II	SPEA2	MOBBO	MOPSO	MOABC
	$R_{H}$	1.009	1.084	1.608	1.323	1.695
1	$C_{V}$	12.5%	85.7%	100%	88.9%	100%
	$C_{V}^{\prime }$	0%	0%	0%	0%	0%
	$R_{H}$	1.017	1.151	1.975	1.663	2.116
2	$C_{V}$	22.2%	62.5%	100%	100%	100%
	$C_{V}^{\prime }$	0%	0%	0%	0%	0%
	$R_{H}$	1.258	1.346	1.715	3.106	2.884
3	$C_{V}$	78.8%	88.9%	100%	100%	100%
	$C_{V}^{\prime }$	0%	0%	0%	0%	0%
	$R_{H}$	1.497	1.609	1.785	3.281	3.329
4	$C_{V}$	100%	100%	100%	100%	100%
	$C_{V}^{\prime }$	0%	0%	0%	0%	0%

## Abbreviations

The following abbreviations are used in this manuscript:

CT	computed tomography
COVID-19	novel coronavirus pneumonia
NSGA-II	Improved fast non-dominated genetic algorithm
MOWWO	multi-objective water wave optimization
MOBBO	Multi-objective biogeography-based optimization
MOPSO	Multi-objective particle swam optimization
MOABC	Multi-objective artificial bee colony
SPEA2	Strength Pareto evolutionary algorithm
WWO	water wave optimization
